# Is first-line antimicrobial therapy still adequate to treat MRSA in the ICU? A report from a highly endemic country

**DOI:** 10.1186/s13054-016-1430-2

**Published:** 2016-08-27

**Authors:** Matteo Bassetti, Elda Righi, Maddalena Peghin, Alessia Carnelutti, Filippo Ansaldi, Cecilia Trucchi, Cristiano Alicino, Enrico Maria Tricarichi, Paola Del Giacomo, Mario Tumbarello

**Affiliations:** 1 Infectious Diseases Division, Santa Maria Misericordia University Hospital, Udine, Italy; 2 IRCCS AOU San Martino IST, Department of Health Sciences, University of Genoa, Genoa, Italy; 3 Institute of Infectious Diseases, Catholic University of the Sacred Heart, Rome, Italy; 4 Clinica Malattie Infettive, Azienda Ospedaliero-Universitaria “Santa Maria della Misericordia”, Piazzale S. Maria della Misericordia, n. 15, 33100 Udine, Italy

Methicillin-resistant *Staphylococcus aureus* (MRSA) infections cause great concern in intensive care units (ICUs) [1]. Although strict infection control protocols have reduced staphylococcal colonization, the ICU still represents a *reservoir* for MRSA infections, playing a role in their circulation to multiple wards and hospitals [2–4]. In critically ill patients, lack of adequate treatment may lead to increased mortality [1]. For this reason, broad-spectrum antimicrobial therapy is often justified among critically ill patients.

We retrospectively analyzed the characteristics of *S. aureus* bloodstream infections (SA-BSI) from two Italian University hospitals during 2010–2014. A total of 17/337 (5 %) were ICU patients; of these, 16 (94 %) had MRSA-BSI compared with 36 % (116/320) from other wards (*P* < 0.001). Lower adequate first-line therapy (defined as therapy administered within 48 h of the positive blood culture and effective against a susceptible pathogen) and infectious diseases (ID) specialist consultation were documented in ICU versus non-ICU patients (18 versus 60 % and 53 versus 24 %, *P* < 0.001 and *P* = 0.02, respectively). When only MRSA infections were considered, adequate therapy and ID consultation remained more common in non-ICU patients (Table 1; difference not significant). Inadequate therapy for non-ICU and ICU patients was mainly associated with beta-lactam use (62/104 versus 10/12, respectively, *P* = 0.19). Patients with MRSA infections in the ICU displayed a lower Charlson score, longer hospitalizations, higher rates of nosocomial infections, endocarditis, and central venous catheter (CVC) and urinary catheter placement. Source control, including CVC removal, was significantly higher in ICU versus non-ICU patients (Table 1). A stepwise logistic regression analysis identified ICU stay (odds ratio (OR) 19.5, 95 % confidence interval (CI) 3.4–384.2, *P* < 0.001), presence of intravascular devices other than CVCs for over 72 h (OR 3.5, 95 % CI 1–13.2, *P* = 0.04), and pulmonary source of infection (OR 3.2, 95 % CI 1.2–9.4, *P* = 0.02) as factors associated with MRSA-BSI. Overall crude 7- and 30-day mortality was similar for MRSA- (Table 1) and SA-BSI (13 versus 18 % in non-ICU and 25 versus 29 % in ICU patients, respectively). Multivariate analysis identified as independent factors for 7-day mortality among patients with MRSA an inadequate targeted treatment (OR 0.19, 95 % CI 0.04–0.86, *P* = 0.03), absence of ID consultation (OR 0.17, 95 % CI 0.04–0.6, *P* = 0.004), and occurrence of endocarditis (OR 4.8, 95 % CI 1.4–17.5, *P* = 0.01) or septic shock (OR 15.9, 95 % CI 4.6– 66.7, *P* < 0.001). High Charlson score (OR 1.25, 95 % CI 1.1–1.5, *P* = 0.004) and septic shock (OR 2.8, 95 % CI 1.0–7.9, *P* = 0.04) were significantly associated with 30-day mortality.

**Table 1:** Characteristics of MRSA bloodstream infections in patients hospitalized in the ICU compared with other wards

Characteristic	Non-ICU (*n* = 116)	ICU (*n* = 16)	*P* value
Age, years (median, IQR)	70.5 (56–77)	59 (56–68)	0.07
Males (%)	82/116 (70.7)	10/16 (62.5)	0.50
Charlson score (median, IQR)	6 (3–7)	2 (1–4)	<0.001
CVC (>72 h) (%)	58/116 (50)	12/16 (75)	0.05
Other intravascular devices (>72 h) (%)	8/116 (6.9)	2/16 (12.5)	0.35
Urinary catheter (>72 h) (%)	40/116 (34.5)	14/16 (87.5)	<0.001
Antimicrobial therapy (<30 days) (%)	50/116 (43.1)	10/16 (62.5)	0.14
Source of infection			
Unknown	46/116 (39.6)	0/16 (0)	0.002
CVC	12/116 (10.3)	3/16 (18.8)	0.53
Pulmonary	12/116 (10.3)	1/16 (6.3)	1.00
Endocarditis	15/116 (12.9)	8/16 (50)	0.001
Skin and soft tissue	12/116 (10.3)	3/16 (18.7)	0.53
Other	19/116 (4.3)	1/16 (6.3)	1.00
CVC removal (%)	42/58 (72.4)	12/12 (100)	0.06
Source control (%)	22/70 (31.4)	12/16 (75)	0.003
Hospitalization, days (median, IQR)	30 (18–44)	56 (25–116)	0.01
Acquisition (%)			
Community acquired	2/116 (1.7)	0/16 (0)	1.00
Health-care associated	40/116 (34.5)	0/16 (0)	<0.001
Hospital-acquired	74/116 (63.8)	16/16 (100)	0.003
Septic shock	22/116 (19)	1/16 (6.3)	0.37
Infectious disease consultation (%)	53/116 (45.7)	4/16 (25)	0.11
Empirical antimicrobial therapy (%)	110/116 (94.8)	16/16 (100)	1.00
Daptomycin	7/104 (6.7)	0/12 (0)	0.35
Glycopeptides	24/104 (23.1)	1/12 (8.3)	0.46
Therapy duration, days (median, IQR)	16 (7-22)	18 (14-27)	0.31
Adequate initial therapy (%)	30/116 (25.9)	2/16 (12.5)	0.39
7-day mortality (%)	27/116 (23.3)	3/16 (18.8)	0.69
30-day mortality	42/116 (36.2)	5/16 (31.3)	0.7

Values are expressed as percentage and median (25th and 75th percentile)

*CVC* central venous catheter, *IQR* interquartile range

The prevalence of MRSA varies widely by geographic region [5]. In our report, overall MRSA rates were comparable to those reported in Italy (33.6 %) by the European Antimicrobial Resistance Surveillance (EARS) in 2014 [5].

Our data highlight that inadequate MRSA first-line therapy can occur in clinical settings known to be at high risk for multi-drug resistant (MDR) infections. Low ID involvement and a priority towards multidrug-resistant Gram-negatives are possible reasons for a reduced first-line use of anti-MRSA compounds in the ICU. Although ICU patients displayed higher rates of inadequate first-line therapy and risk factors associated with increased mortality (e.g., reduced ID consultation and endocarditis) [6], overall mortality was comparable between groups. This may be related to a limited number of patients with septic shock in the ICU group; furthermore, low Charlson scores and higher source control in the ICU group compared with the non-ICU group may have contributed to achieve positive outcomes.

In conclusion, our study draws attention to an alarming proportion of first-line inadequate therapy among patients with SA-BSI in the ICU. In this setting, the use of protocols including anti-MRSA agents in patients at risk for Staphylococcus aureus bacteremia (SAB) should be recommended, and clinicians must retain a high level of suspicion for MRSA infections in order to select an appropriate early antimicrobial treatment and ultimately reduce mortality.

## References

[1] Klevens RM, Morrison MA, Nadle J (2007). Invasive methicillin-resistant Staphylococcus aureus infections in the United States. JAMA..

[2] Mitchell BG, Collignon PJ, McCann R (2014). A major reduction in hospital-onset Staphylococcus aureus bacteremia in Australia-12 years of progress: an observational study. Clin Infect Dis.

[3] Stenehjem E, Stafford C, Rimland D (2013). Reduction of methicillin-resistant Staphylococcus aureus infection among veterans in Atlanta. Infect Control Hosp Epidemiol.

[4] Hoefnagels-Schuermans A, Borremans A, Peetermans W (1997). Origin and transmission of methicillin resistant Staphylococcus aureus in an endemic situation: differences between geriatric and intensive care patients. J Hosp Infect..

[5] European Centre for Disease Prevention and Control (ECDC). Antimicrobial resistance surveillance in Europe. 2014. http://ecdc.europa.eu/en/publications/Publications/antimicrobial-resistance-europe-2014.pdf. Accessed 31 July 2016.

[6] Vogel M, Schmitz RP, Hagel S (2016). Infectious disease consultation for Staphylococcus aureus bacteremia--a systematic review and meta-analysis. J Infect.

